# FNDC5/Irisin: Physiology and Pathophysiology

**DOI:** 10.3390/molecules27031118

**Published:** 2022-02-08

**Authors:** Rashid Waseem, Anas Shamsi, Taj Mohammad, Md. Imtaiyaz Hassan, Syed Naqui Kazim, Anis Ahmad Chaudhary, Hassan Ahmed Rudayni, Mohammed Al-Zharani, Faizan Ahmad, Asimul Islam

**Affiliations:** 1 Centre for Interdisciplinary Research in Basic Sciences, Jamia Millia Islamia, New Delhi 110025, India; rashid.waseem439@gmail.com (R.W.); anas.shamsi18@gmail.com (A.S.); taj144796@st.jmi.ac.in (T.M.); mihassan@jmi.ac.in (M.I.H.); skazim@jmi.ac.in (S.N.K.); 2 Department of Biology, College of Science, Imam Mohammad Ibn Saud Islamic University, Riyadh 11564, Saudi Arabia; aachaudhary@imamu.edu.sa (A.A.C.); harudayni@imamu.edu.sa (H.A.R.); mmylzahrani@imamu.edu.sa (M.A.-Z.); 3 Department of Biochemistry, Jamia Hamdard, New Delhi 110062, India; fahmad@jmi.ac.in

**Keywords:** irisin, structural insight, therapeutic potential, human pathophysiology

## Abstract

A sedentary lifestyle or lack of physical activity increases the risk of different diseases, including obesity, diabetes, heart diseases, certain types of cancers, and some neurological diseases. Physical exercise helps improve quality of life and reduces the risk of many diseases. Irisin, a hormone induced by exercise, is a fragmented product of FNDC5 (a cell membrane protein) and acts as a linkage between muscles and other tissues. Over the past decade, it has become clear that irisin is a molecular mimic of exercise and shows various beneficial effects, such as browning of adipocytes, modulation of metabolic processes, regulation of bone metabolism, and functioning of the nervous system. Irisin has a role in carcinogenesis; numerous studies have shown its impact on migration, invasion, and proliferation of cancer cells. The receptor of irisin is not completely known; however, in some tissues it probably acts via a specific class of integrin receptors. Here, we review research from the past decade that has identified irisin as a potential therapeutic agent in the prevention or treatment of various metabolic-related and other diseases. This article delineates structural and biochemical aspects of irisin and provides an insight into the role of irisin in different pathological conditions.

## 1. Introduction

Myocytes or muscle cells produce myokines (which are cytokines) in response to the contraction of muscles. Myokines are involved in the autocrine regulations for muscle metabolism, whereas in other tissues and organs such as adipose tissue, the brain, and the liver, they are responsible for paracrine or endocrine regulation through their receptors [1]. Myokine receptors are present in muscle, heart, liver, fat, pancreas, bone, immune, and brain cells. The receptor’s location indicates that myokines have multiple functions [2]. In addition to several other myokines which are found in skeletal muscles such as IL-15, IL-6, IL-8, leukemia inhibitory factor, FGF21, BDNF, etc., there is a newly discovered myokine, irisin, which was reported for the first time by Bostrom et al. in 2012 [3]. It was reported to be a molecule capable of inducing changes in adipose tissues and activating thermogenesis [3]. It was named ‘irisin’ after Iris, the Greek messenger goddess [3]. Since its discovery, irisin has been the subject of extensive research due to its function in various physiological conditions.

Irisin is derived from the FNDC5 protein through its extracellular fragment proteolytic cleavage and secreted in the peripheral circulation [4]. Earlier studies show that irisin converts WAT (white adipose tissue) into BAT (brown adipose tissue) mainly by upregulating the UCP1 expression through enhanced energy expenditure [3]. Recently, it was revealed that irisin is involved in improving glucose tolerance and helping in ameliorating insulin resistance [5].

Various studies have discovered important biological roles of irisin, such as in the regulation of depressive behavior [6], proliferating osteoblasts [7], and cortical bone mass [8]. It was suggested that irisin also has some beneficial roles in the central nervous system [9,10], and that it activates the Akt and ERK1/2 signaling pathways in brain tissues [9]. Moreover, irisin regulates some risk factors of AD (Alzheimer’s disease) [11], which includes altered neurogenesis, oxidative stress, insulin resistance, and imbalance of neurotrophic factors. Irisin has been found to have an important role in various conditions, including hippocampal neurogenesis, inflammation, aging, and other metabolic conditions [12,13]. Since there are various physiological effects of irisin in the body, it has gained interest from researchers all over the world and has been studied extensively. This review focuses on irisin’s structure, discovery, expression, functions, and its role as an exercise-induced hormone.

## 2. Irisin: Biosynthesis, Structure, and Downstream Signaling

### 2.1. Biosynthesis and Secretion of Irisin

Irisin is mainly secreted from skeletal muscles. However, immunohistochemical studies have shown that it is also found in the pancreas, testes, liver, and stomach [4]. Irisin secretion and synthesis are induced by exercise and PGC1α [13]. PGC1-α is a multispecific coactivator of transcription, which is competent in multiple gene regulation in response to the nutritional and the physiological signals in tissues such as brown adipose tissue, skeletal muscle, and heart and liver tissue [14]. As irisin is an exercise-induced myokine, the circulating level of irisin increases in individuals engaged in exercise-induced activities and progressively decreases in those who are sedentary and less active [15]. Prolonged exercise increases PGC1α expression mainly in the skeletal muscles and heart and improves various metabolic parameters, including AMPK activation, PGC1α phosphorylation, insulin sensitivity and signaling, and FNDC5 production, followed by the cleavage of FNDC5 to secrete irisin [15,16].

A comparative study on irisin has shown 100% identity between murine and human irisin sequences) [3]. Conversely, it has been found that in the human FNDC5 gene, there is an unusual ATA start codon [17] which was previously identified as a null mutation, and it has been suggested that in humans, this mutation would prevent irisin production and release in the blood [18]. However, in humans, FNDC5 made from the ATA-FNDC5 sequence was detected, proving that it is not a pseudogene [18]; however, it was suggested to be in the category of genes that have lost their protein-coding ability [19]. Moreover, it has already been proven that human irisin is mainly translated from its non-canonical start codon [17].

There was also a concern about the lack of specificity in anti-irisin antibodies [20]. There were contradictory remarks on the existence of irisin by experimental evidence, but many sensitive approaches—including ELISA assays and quantitative mass spectrometry— have been employed successfully to confirm irisin’s identity and to measure the circulating level of irisin in humans [17,21,22,23]. Lee et al. employed mass spectrometry for the determination of the identity of FNDC5-immunoreactive bands detectable in human serum [21]. The mass spectrometry analysis identified a unique peptide mapped to the known sequence of irisin, which validated the immunoblot identification of circulating irisin in humans [21]. Later, another study employing mass spectrometry identified and quantitated human irisin in plasma [17]. In the study, human irisin was identified and quantitated in plasma using mass spectrometry with control peptides enriched with heavy stable isotopes as internal standards. [17]. In line with this, it was demonstrated that cold exposure increases circulating irisin levels in humans, suggesting that exercise-induced irisin could have evolved from shivering-related muscle contraction [21]. Recently, Colaianni et al. detected decreased serum irisin levels in patients with age-related bone diseases in comparison to healthy subjects [24]. Moreover, many studies have confirmed that circulating irisin levels in the body are affected by several factors, such as diet, metabolic diseases, and other pathological disorders [4,25,26]. These data support the claim that irisin does exist and is regulated by exercise.

### 2.2. Structural Features and Signaling Pathways

**Figure 1:**
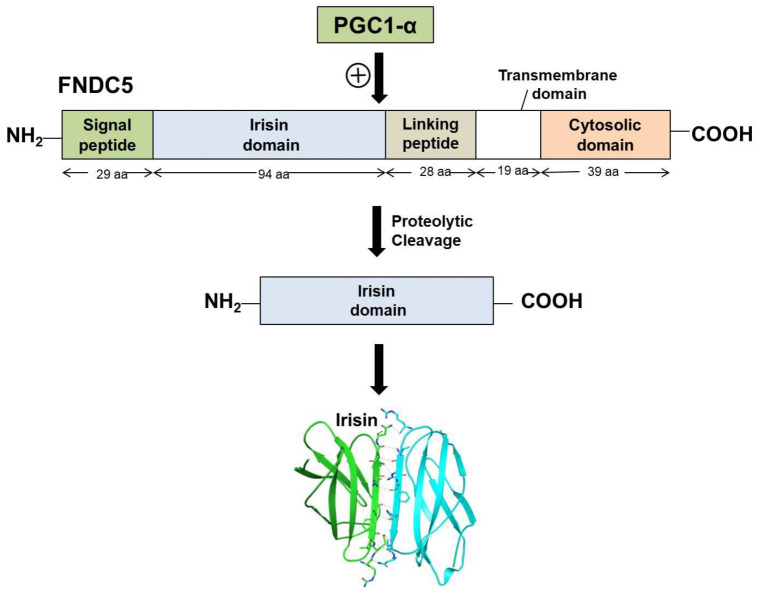
Schematic representation of FNDC5 structure and formation of irisin.

Irisin is a portion of the cell membrane protein known as FNDC5 [14]. FNDC5 consists of a signal peptide, a fibronectin III domain, and a C-terminal domain. FNDC5 comprises 209 amino acid residues, having a signal sequence of 29 amino acids at the N-terminal end, followed by a 94-amino-acid residue fibronectin III (FNIII) 2 domains (irisin domain), a linking peptide comprising 28 amino acid residues, a 19-amino-acid residue transmembrane domain, and a cytoplasmic domain consisting of 39 amino acid residues. The biochemical and crystallographic studies have shown that irisin exists as a homodimer, with the continuous β-sheet interactions forming the core of the dimer. The crystal structure of irisin revealed that it contains a fold which is similar to FNIII proteins. The first study which reported the crystal data of irisin shows that irisin structure is homologous to FNIII domains, as it consists of an N-terminal domain (residues 30–123) along with a C-terminal tail composed of residues 124–140 which is mostly disordered [27]. Although all of the FNIII domains have limited homology and share only 15–20% sequence identity, their structures have surprisingly similar folds, comprising a β-sandwich with four β-strands on one side and three on the other [27]. Unlike other FNIII structures, irisin constitutes a continuous inter-subunit β-sheet dimer, which has an essential implication for receptor activation and signaling. The core of the irisin dimer is formed by continuous β-sheet interactions and 10 backbone hydrogen-bonds between the two interacting four-stranded β-sheets. Hence, the irisin structure unveils the first instance of a continuous β-sheet dimer made between two FNIII domains. Irisin is a 112-amino-acid peptide that includes the 94-amino-acid residue extracellular FNIII domain, cleaved from the C-terminal end of FNDC5. Figure 1 depicts the schematic representation of the structure of FNDC5 and the formation of irisin through its proteolytic cleavage.

Glycosylation is a very common post-translational modification of proteins where the attachment of carbohydrates leads to greater heterogeneity in the structure of glycans. Oligosaccharides influence the protein’s physicochemical properties, which are essential to obtain accurate protein conformation and protect against proteolysis, and are also essential for their biological function in diverse metabolic processes [28]. There are two N-glycosylation sites in irisin at the Asn-7 and Asn-52 positions [29]. The molecular weight of FNDC5 proteins ranges from 20 to 32 kDa, depending on the number and structure of glycan moiety attached to the molecule of protein during the process of post-translational modification [4]. Deglycosylation lowers the molecular weight of irisin to 12–15 kDa [11]. In some studies, it is shown that post-translational modification, i.e., N-glycosylation, has an important role in irisin activity. Both glycosylated and nonglycosylated forms of irisin have been used in a recent study [5] and further research is required to determine the glycosylation pattern and effects of the glycosylation of irisin in various physiological conditions.

There are several intracellular signaling pathways through which FNDC5/irisin elicits its biological functions. The major pathways through which irisin exert its action in white adipocytes browning, neural differentiation, and osteoblast proliferation, are MAPK signaling pathways. In addition to this, there are some other signaling cascades such as the AMPK pathway, PI3K/AKT, and STAT3/Snail pathway, which mediate some other important functions of FNDC5/Irisin [30].

**Figure 2:**
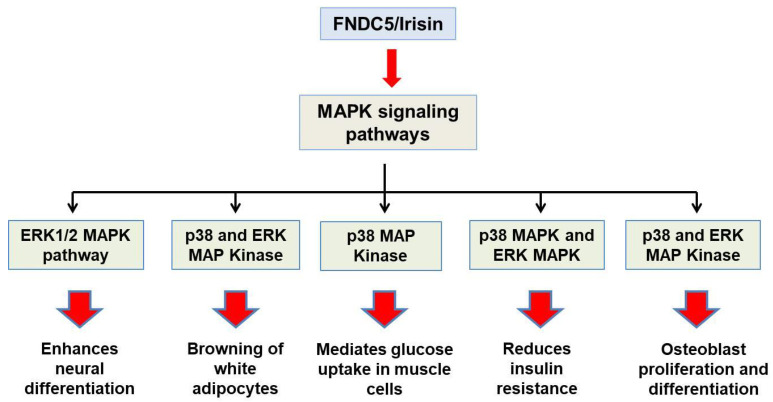
Schematic representation of physiological roles of Fndc5/irisin through MAPK signaling pathways.

**Figure 3:**
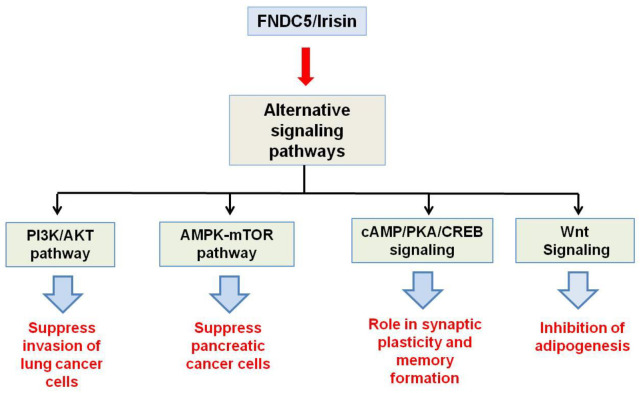
Schematic representation of physiological activities of Fndc5/irisin through pathways other than MAP Kinase signaling.

Major functions which the *fndc5/irisin* gene elicits in the body are mediated by p38 and ERK1/2 signaling. WAT browning is induced by irisin through p38 and ERK. It was shown both in vivo and in vitro that recombinant irisin treatment increases levels of phosphorylated p38 as well as phosphorylated ERK, which in turn results in the upregulation of the UCP1 expression level [29]. Irisin through p38 MAPK and ERK1/2 signaling is not only responsible for the browning of WAT but also induces neural cell differentiation, osteocyte proliferation, glucose uptake by the muscles, and a reduction in insulin resistance [30]. The main physiological effects which irisin shows through MAPK signaling pathways are depicted in Figure 2. AMPK and PI3K/AKT pathways mediate the effect of irisin in proliferation, anti-inflammatory, and anti-metastatic activities. A report showed that irisin enhances the proliferation of H19-7 cells through STAT3 signaling instead of AMPK and/or ERK, so it can be inferred that irisin exerts its neuroprotective effect partly through STAT3 signaling [30]. It was demonstrated that irisin treatment activates the AMPK pathway and downregulates the mTOR pathway, thereby suppressing pancreatic cancer cell growth, and thus inhibits the epithelial–mesenchyme transition (EMT) of pancreatic cancer cells [31]. Irisin has also been shown to mediate its effect through the PI3/AKT pathway in lung cancers. A study showed that irisin can reduce the expression of the EMT marker and inhibits the Snail expression via PI3K/AKT pathway, thereby inhibiting the invasion, migration, and proliferation of lung cancer cells [32]. Irisin has also been found to stimulate the cAMP/PKA/CREB pathway, thereby regulating neuronal plasticity and preventing memory impairment [33]. It was demonstrated that irisin can inhibit adipogenesis through activation of the Wnt expression and following the repression of transcription factors [34]. In Figure 3, the role of irisin has been shown in different physiological conditions through pathways other than MAPK signaling.

### 2.3. Irisin Receptor

At present, the receptor for irisin has not been fully identified; however, in a recent study, Kim et al. suggested that the αV family of integrin receptors are likely irisin receptors in thermogenic fats and osteocytes [35]. In the study, quantitative proteomic analysis in MLO-Y4 osteocytes showed that irisin binds efficiently to the integrin β1-α1 heterodimers. The protein–protein binding assay was performed to check the binding affinity between irisin and several integrin complexes [35]. It was found that most integrin complexes, including integrin β1-α1, showed significant binding with irisin; however, αV/β5 integrins showed the highest binding affinity. HDX-MS also demonstrated that irisin binds to αV/β5 integrins which allow the mapping of binding motifs on irisin and integrin complexes. Further, it was demonstrated that a very low concentration (10 pM) of irisin treatment resulted in the activation of the classic integrin signaling pathway in MLO-Y4 osteocytes [35]. Moreover, it was revealed that when the αV integrins are chemically inhibited, the signaling and function of irisin in osteocytes and fat cells are blocked [35]. Taken together, all these data suggest that although no specific receptor of irisin has been identified yet, it exerts its action via αV/β5 integrins in bone and fat tissues. Conversely, these specific effects of irisin via interaction with αV/β5 are not completely understood in vivo, either due to the ability of αV/β5 to interact with other ligands, or the binding affinity of irisin with other integrin complexes [36]. Although αV/β5 integrins have been shown as irisin receptors in some tissues, there is also a possibility of other receptors both within and outside of the integrin family.

## 3. The Role of Irisin in Human Pathophysiology

The most significant roles of irisin include browning of white adipocytes, neural proliferation, and bone metabolism [3,37,38]. Other effects of irisin involve glucose homeostasis and cardiac metabolism, which are still under investigation [4]. Here, we discuss irisin’s role in some major physiological and pathophysiological conditions.

### 3.1. Irisin in Obesity and Diabetes

Obesity results from persistent positive energy balance, which occurs when the intake of energy is higher than the expenditure of energy. It is associated with the risk of life-threatening diseases such as type 2 diabetes, stroke, heart diseases, etc. Fat accumulation in adipose tissue is important for energy storage and to insulate the body. However, the excessive accumulation of body fat leads to obesity. Adipose tissues are the main organ for fat storage and have a fundamental role in metabolism [39]. Based on structure and function, adipose tissues were distinguished as WAT and BAT. WAT consists of mainly mature white adipocytes with a nucleus that is peripherally located and a big single lipid droplet. BAT is morphologically different from WAT because BAT has a centrally located nucleus, numerous small lipid droplets, and many mitochondria. Lipids present in BAT are used primarily for heat generation and oxidative phosphorylation [4]. WAT stores energy in the form of triglyceride and releases free fatty acids when needed, whereas BAT burns fat to maintain temperature by a process called non-shivering thermogenesis [40]. The thermogenic capacity (ability to generate heat) depends upon the UCP1 which forms a pore in the inner mitochondrial membrane. Due to this, leakage of protons occurs, which dissipates the electrochemical proton gradient in the mitochondrial matrix that is required for ATP synthesis. This therefore results in blunted ATP synthesis and the release of energy as heat. Overexpression of UCP1 in WAT is suggested as the therapy for preventing obesity.

**Figure 4:**
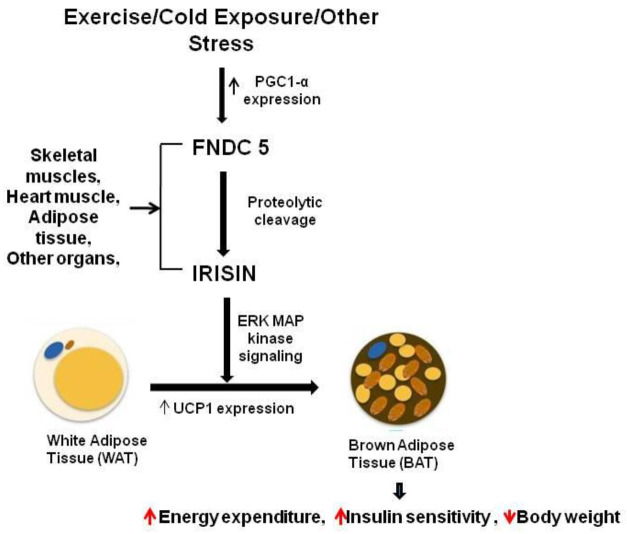
Irisin secretion and its role in fat browning.

Zhang et al. demonstrated that irisin also affects WAT’s functioning, and the effects of its activity are dependent on the degree of cell differentiation [29]. In vitro studies used mature adipocytes, and undifferentiated preadipocytes, to assess the effect of irisin. Irisin induces the UCP-1 expression levels in mature fat cells, which results in reprogramming WAT to take on the phenotype of BAT by the process of fat browning [4]. Expression of the browning-associated genes and UCP1 protein is upregulated by irisin in the fresh adipose tissues, as well as in cultured primary mature adipocytes. It was observed that treating human subcutaneous WAT with irisin increases the expression of UCP1 by activating p38 MAPK and ERK signaling [3]. To confirm this, specific inhibitors were used for blocking either of these two pathways, and it was revealed that this causes the abolition of irisin-induced UCP1 upregulation [29]. Therefore, it was concluded that WAT browning is induced by irisin through p38MAPK and ERK MAPK signaling [29]. A recent study demonstrated that a lack of irisin is coupled with a poor browning response and glucose/lipid derangement [41]. It can thus be concluded that the ability of irisin to convert WAT cells into the phenotype of BAT cells can be a potential therapeutic target for obesity and other associated diseases. In Figure 4, we show the secretion of irisin from FNDC5 as a mature peptide and its role in obesity through fat browning.

Irisin acts as an insulin-sensitizing hormone, and it is believed that irisin improves hepatic glucose and lipid metabolism by promoting pancreatic β cell functions and helps in the amelioration of insulin resistance and type 2 diabetes [42,43]. Irisin facilitates the uptake of glucose by skeletal muscles, and also improves lipid metabolism and hepatic glucose. It shows a positive effect on hyperglycemia and hyperlipidemia caused by metabolic syndrome and obesity [44]. There is an inverse association between irisin and type 2 diabetes as shown by Choi et al. in a study, where reduced irisin concentrations were reported in diabetic patients compared to the control [45]. In line with this, another study reported significantly decreased irisin concentrations in adults with T2DM regardless of age, gender, or BMI [46]. In diabetic patients, vascular complications resulting from endothelial dysfunction are the major causes of death [47]. In type 2 diabetes, irisin has been found to alleviate endothelial dysfunction partially via reducing oxidative/nitrative stresses through inhibition of signaling pathways, implicating NF-κB/iNOS and PKC-β/NADPH oxidase [47]. These studies altogether suggest that irisin may be a potential agent for the treatment of diabetic complications.

### 3.2. Irisin in the Nervous System

Physical exercise shows beneficial effects on the functioning of the nervous system. Moderate and regular exercise enhances the differentiation and proliferation of mouse neurons, increases the survival period, and stimulates migration [48]. Exercise ameliorates negative outcomes in neurological diseases, since exercise has many positive effects on the nervous system. It was expected that exercise-induced hormone irisin would also have some beneficial influences. Irisin is found in cerebral Purkinje cells, hypothalamus, and cerebrospinal fluid, and plays some essential roles in the central nervous system [10,49]. Various evidence showed that irisin crosses the blood–brain barrier, from where it induces BDNF, which is involved in regulating synaptic plasticity [36,50]. Earlier, it was reported that irisin enhances cell proliferation in H19-7 HN cells of mice [51]. Moreover, irisin plays a crucial role in activating autophagy and thus exhibits a protective role against inflammation [52]. Several reports are investigating the protective roles of irisin through activation of autophagy as an anti-inflammatory strategy [52,53].

A recent study demonstrated that during cerebral ischemia-reperfusion, irisin regulates BDNF expression in the rodent stroke model, which indicates that irisin confers its beneficial effect through BDNF in ischemic stroke [54]. Recently, it was reported that irisin inhibits the expression and activity of MMP-9 in brain tissues and thereby protects the blood–brain barrier from ischemic stroke [55]. Moreover, it has been demonstrated that during ischemic stroke, irisin exerts its beneficial role by inhibiting ROS-NLRP3 signaling in vivo [56] and through suppression of TLR4/MYD88 signaling pathways in vitro [57]. Irisin alleviates the neuronal apoptosis and post-ischemic inflammation, and improves neurological dysfunction through activation of the notch signaling pathway [58]. In another study, it was reported that in the case of cerebral ischemia, irisin treatment could reduce brain infarct volume, brain edema, neurological deficits, and the decline in body weight [59]. Further, it was suggested that irisin reduces ischemia-induced neuronal injury through activation of the Akt and ERK1/2 signaling pathways. It was demonstrated that the activation of ERK1/2 and Akt might be essential for the neuroprotective effects of irisin, as inhibiting ERK1/2 and Akt pathways through specific chemical inhibitors abolished the neuroprotective effects shown by irisin [59]. These studies give us an idea about the contribution of irisin in neuroprotection by physical exercise in cerebral ischemia. It is therefore a potential agent for the prevention and treatment of ischemic stroke. In addition to BDNF, irisin regulates some other factors which affect hippocampal proliferation, such as neurogenesis-related STAT3 signaling [60]. Several AD risk factors are modulated by irisin, including oxidative stress, insulin resistance, imbalance of neurotrophic factors, and impaired neurogenesis. Irisin exhibits protective actions against the aberrant expression of synapse-related genes, therefore indicating its potential role in attenuation of memory and synaptic impairment in AD models [33]. It is established that FNDC5/irisin is a novel mediator of the positive effects of exercise on memory and synapse function. Hence, it can be proposed that either pharmacologically or through exercise, boosting brain FNDC5/irisin levels may constitute a novel therapeutic strategy for protection against synaptic dysfunction or prevention from memory impairment in AD [33].

Deposition of Amyloid β peptides (Aβ40 and Aβ42) is detected at the early stages in AD pathology, and preventing this deposition could assist in the cure of AD at initial stages [61]. It was observed that overexpression of FNDC5/irisin could significantly decrease the level of both Aβ40 and Aβ42 secretion in the media. It was investigated that FNDC5/irisin affects Aβ metabolism by interacting with amyloid precursor protein (APP). In FNDC5/irisin, the main binding domain of APP is localized between the 1–16 amino acids at the N-terminal of the Aβ sequence. It was observed that suppressing the interaction between these two molecules by Aβ_1–16_ peptide treatment results in increased Aβ_40_ and Aβ_42_ secretion compared to the control condition, where Aβ_1–16_ peptide was not treated. Therefore, it can be concluded that FNDC5 binds with the Aβ N-terminal sequence and affects the β-cleavage of APP, which in turn helps reduce Aβ production [62]. A deeper knowledge of the mechanisms by which the interaction between FNDC5/irisin and APP might affect the production of Aβ in an exercise-dependent manner may offer a new preventive strategy against AD development.

### 3.3. Irisin in Bone Metabolism

The loss of bone mass with age increases the susceptibility to fractures, and hence is implicated in medicine and socioeconomics. Osteoporosis increases comorbidities, reduces life quality, impairs mobility, and shortens lifespan [63]. Recent studies on mice showed that irisin directly targets the bone tissue, and treatment of recombinant irisin increases cortical mineral density and improves geometry and bone strength [8]. In the bone marrow, stromal cells can be more efficiently differentiated into mature osteoblasts by irisin administration [64]. The treatment of irisin increases the level of plasma sclerostin (a specific product of osteocyte which causes bone resorption and initiates the remodeling of bone), as well as mRNA levels of sclerostin in the osteocyte culture in a dose-dependent manner [35]. All these results demonstrate that irisin could protect osteocytes against apoptosis in vitro and induce the sclerostin expression levels in vivo [35].

In vitro studies suggest that irisin promotes the proliferation of osteoblasts and raises the expression levels of osteoblast transcriptional regulators such as osterix/sp7, Runt-related transcription factor-2, and osteoblast differentiation markers including alkaline phosphatase, collagen type 1 alpha-1, osteopontin, and osteocalcin [38]. Additionally, irisin can increase the calcium deposition and activity of alkaline phosphatase in cultured osteoblasts [38]. Irisin exerts its osteogenic effects through activation of the ERK and p38 MAPK pathways. This was supported by the study that inhibiting these pathways with specific inhibitors resulted in diminishing irisin’s upregulatory effects on the activity of alkaline phosphatase and Runx2 expression. It is therefore suggested that irisin targets osteoblasts directly, promoting osteoblast differentiation and proliferation by activating the P38/ERK MAPK signaling cascade in vitro [38], and these findings were in accordance with Colaianni’s recent study showing that irisin promotes the osteogenic differentiation of bone marrow stromal cells through the ERK signaling pathway [8]. In addition to stimulating bone remodeling, irisin also functions as a counter-regulatory hormone, since it acts directly on osteoclast progenitors to enhance differentiation and promote bone resorption [65]. It was revealed by RNA sequence that irisin stimulates differential gene expression, which includes upregulating markers for resorption and differentiation of osteoclasts [65].

The clinical trials of irisin indicate a positive effect on bone formation. Serbest et al. reported that the level of irisin in the blood increased in the fracture union process, and as irisin receptors were in human bone tissue, fracture union was affected [66]. In another study, it was found that there was a lower irisin level in female athletes (without menstruation) compared to eumenorrheic athletes and non-athletes [67]. Moreover, in all of the athletes, there was a positive correlation between irisin concentration and volumetric bone mineral density (BMD) [67]. More recently, irisin deficiency has been shown to cause disturbance in bone metabolism [68]. In osteoblast lineage, FNDC5/irisin was deleted, and thus FNDC5/irisin KO mice were generated. There was a remarkable decrease in irisin expression at both gene and protein levels in bones, causing lower bone density and much-delayed bone development [68]. Regardless of the raised doubts and controversies, scientists suggest that irisin is a novel key player in the metabolism of bone, and it is emerging as a potential therapeutic agent for the treatment of bone diseases. Moreover, an extension of these findings on humans would encourage the use of irisin as a therapeutic agent for the treatment and prevention of sarcopenia, osteoporosis, and other bone-associated diseases.

### 3.4. Irisin in Carcinogenesis

Irisin is also implicated in carcinogenesis, but its role in the progression of cancer is currently ambiguous. It is generally known that obesity is a risk factor for various cancers. Since irisin is a potential therapeutic agent against obesity, many researchers focus on studying the link between various cancers and irisin. While searching the role of irisin in cancer, malignant breast epithelial cells (MCF-7), human non-malignant breast epithelial cells (MCF-10a), and malignant aggressive breast epithelial cells (MDA-MB-231) were used for a study, and it was found that the number of malignant breast tumor cells decreased significantly upon exposure to irisin [69]. However, it was difficult to infer whether the reduced cell number is a result of decreased cell proliferation or increased apoptotic cell death [70]. This indicates that irisin can reverse the hallmark of cancer of resisting cell death [71] via promoting caspase 3 activity and therefore apoptosis. Increased serum irisin levels can reduce the risk of the development of breast cancer by 90%, and patients who develop breast cancer have significantly lower irisin serum levels than healthy individuals [72].

Another study revealed that irisin inhibited the growth of pancreatic cancer (PC) cell lines, Panc03.27, and MIA PaCa-2 significantly in a dose-dependent manner. Irisin activates the AMPK pathway and suppresses mTOR signaling, thereby suppressing PC-cell-growth-inhibiting epithelial–mesenchymal transition (EMT) of PC cells [31].

**Figure 5:**
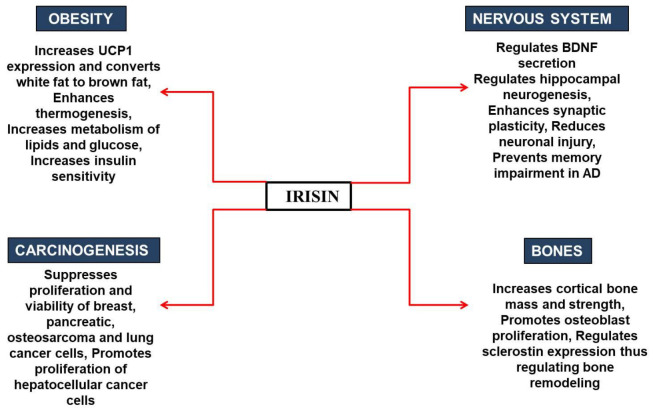
Role of irisin in various physiological conditions.

Various studies have also been conducted to investigate irisin’s role in other cancers [73,74]. It was found that an increase in the level of irisin leads to decreased proliferation, viability, and invasiveness in a lung cancer cell by inhibiting EMT mediated by the PI3K/Akt pathway [32]. It was revealed that irisin could reverse IL-6-induced EMT by inhibiting the STAT3 pathway in osteosarcoma [74]. Irisin has been found to affect the expression of Snail (transcription factor), which is involved in EMT, and inhibits the transcription of the gene encoding E-cadherin, a characteristic of epithelial-derived cells [73]. A report showed that the level of irisin significantly increases in patients with renal tumors, hence suggesting that irisin can be used as a biomarker for renal cancer diagnosis [75]. It is known that a kinase, MARK4, is associated with different cancers and uncontrolled neural migration, and is a potential therapeutic drug target for cancer and AD therapy. On the other hand, irisin is identified as a potential therapeutic agent against AD [33,76]. We have recently shown through a protein–protein interaction study that irisin can inhibit the MARK4 via binding with an excellent affinity and subsequently inhibit its activity [61]. Therefore, irisin can be used as a therapeutic agent against MARK4-directed diseases. When it is known that there is an inverse relationship between cancer and AD and that both share a common biological mechanism [77], exploring irisin as a potential therapeutic agent against these diseases would provide a platform for clinical research against cancer and AD. Although available data suggest that irisin can be a possible substance for the regression of cancer by decreasing pro-inflammatory markers linked to obesity, there are still a few controversies regarding the benefits of irisin, as some studies show no effect of irisin on various cancers [69]. Therefore, a closer inspection is required to understand the direct involvement of irisin in cancer prevention and therapeutics. Figure 5 summarizes the beneficial health effects of irisin in different physiological conditions.

## 4. Conclusions

We reviewed the function of irisin in different tissues. It was suggested that irisin is involved in various diseases such as obesity, type 2 diabetes, osteoporosis, cerebral ischemia, and Alzheimer’s disease. Moreover, it was observed that irisin is also associated with some cancers such as breast cancer, pancreatic cancer, and prostate cancer. It has been known for a long time that physical exercise improves quality of life and can lower the risk of several diseases. The discovery of irisin in 2012 by Bostrom et al. was a breakthrough and demonstrated that irisin is a molecular mimic of exercise. It was revealed for the first time that irisin can elicit beneficial effects of exercise at the molecular level. The main function of irisin has been shown to promote the browning of white adipocytes. The other beneficial physiological effects exerted by irisin include anti-inflammatory properties and associated anti-metastatic effects, neuroprotective effects, and mitigation of oxidative stress [30]. Although various signaling pathways have been elucidated in order to know the action of irisin in pathological conditions, much work is needed to determine its reception mechanism and mode of action in different tissues. Despite the need for further research, irisin remains an interesting molecule from a pathophysiological point of view and is a potential therapeutic target for various metabolic diseases. Since irisin is a multifunctional protein and has beneficial health effects, there is a need for more research to investigate and test the therapeutic applications of this important myokine.

## Data Availability

The information that supports the findings of this study is available in this article.

## Abbreviations

FNDC5	Fibronectin type III domain-containing protein 5
FGF21	Fibroblast growth factor 21
BDNF	Brain-derived neurotrophic factor
WAT	White adipose tissue
BAT	Brown adipose tissue
UCP1	Uncoupling protein 1
PGC1 α	Peroxisome proliferator-activated receptor γ (PPAR-γ) coactivator 1-α
FNDC9	Fibronectin type III domain containing 9
PRDM16	PR domain zinc finger protein 16
AMPK	Adenosine monophosphate-activated protein kinase
PI3K	Phosphatidylinositol 3-kinase
STAT3	Signal transducer and activator of transcription 3
MAPK	Mitogen-activated protein kinase
ERK1/2	Extracellular signal-regulated kinase 1/2
EMT	Epithelial-mesenchyme transition
cAMP	Cyclic adenosine 3, 5- monophosphate
CREB	cAMP response element binding
ATP	Adenosine triphosphate
AD	Alzheimer’s disease
APP	Amyloid precursor protein
ALP	Alkaline phosphatase
mTOR	Mammalian target of rapamycin
MARK4	Microtubule Affinity Regulating Kinase 4

## References

[1] Carson B.P. (2017). The potential role of contraction-induced myokines in the regulation of metabolic function for the prevention and treatment of type 2 diabetes. Front. Endocrinol..

[2] Delezie J., Handschin C. (2018). Endocrine crosstalk between skeletal muscle and the brain. Front. Neurol..

[3] Boström P., Wu J., Jedrychowski M.P., Korde A., Ye L., Lo J.C., Rasbach K.A., Boström E.A., Choi J.H., Long J.Z. (2012). A PGC1-α-dependent myokine that drives brown-fat-like development of white fat and thermogenesis. Nature.

[4] Korta P., Pocheć E., Mazur-Biały A. (2019). Irisin as a multifunctional protein: Implications for health and certain diseases. Medicina.

[5] Li X., Duan H., Liu Q., Umar M., Luo W., Yang X., Zhu J., Li M. (2019). Construction of a Pichia pastoris strain efficiently secreting irisin and assessment of its bioactivity in HepG2 cells. Int. J. Biol. Macromol..

[6] Wang S., Pan J. (2016). Irisin ameliorates depressive-like behaviors in rats by regulating energy metabolism. Biochem. Biophys. Res. Commun..

[7] Chen Z., Zhang Y., Zhao F., Yin C., Yang C., Wang X., Wu Z., Liang S., Li D., Lin X. (2020). Recombinant irisin prevents the reduction of osteoblast differentiation induced by stimulated microgravity through increasing β-catenin expression. Int. J. Mol. Sci..

[8] Colaianni G., Cuscito C., Mongelli T., Pignataro P., Buccoliero C., Liu P., Lu P., Sartini L., di Comite M., Mori G. (2015). The myokine irisin increases cortical bone mass. Proc. Natl. Acad. Sci. USA.

[9] Jodeiri Farshbaf M., Alviña K. (2021). Multiple roles in neuroprotection for the exercise derived myokine Irisin. Front. Aging Neurosci..

[10] Piya M.K., Harte A.L., Sivakumar K., Tripathi G., Voyias P.D., James S., Sabico S., Al-Daghri N.M., Saravanan P., Barber T.M. (2014). The identification of irisin in human cerebrospinal fluid: Influence of adiposity, metabolic markers, and gestational diabetes. Am. J. Physiol. Endocrinol. Metab..

[11] Erickson K.I., Weinstein A.M., Lopez O.L. (2012). Physical activity, brain plasticity, and Alzheimer’s disease. Arch. Med. Res..

[12] Panati K., Suneetha Y., Narala V. (2016). Irisin/FNDC5–An updated review. Eur. Rev. Med. Pharmacol. Sci..

[13] Waseem R., Shamsi A., Mohammad T., Alhumaydhi F.A., Kazim S.N., Hassan M.I., Ahmad F., Islam A. (2021). Multispectroscopic and Molecular Docking Insight into Elucidating the Interaction of Irisin with Rivastigmine Tartrate: A Combinational Therapy Approach to Fight Alzheimer’s Disease. ACS Omega.

[14] Norheim F., Langleite T.M., Hjorth M., Holen T., Kielland A., Stadheim H.K., Gulseth H.L., Birkeland K.I., Jensen J., Drevon C.A. (2014). The effects of acute and chronic exercise on PGC-1α, irisin and browning of subcutaneous adipose tissue in humans. FEBS J..

[15] Clin A. (2018). The Relationship between High-Fat Diet and Fibronectin Type-III Domain-Containing Protein 5 mRNA Expression. Anatol. Clin. J. Med. Sci..

[16] Moreno-Navarrete J.M., Ortega F., Serrano M., Guerra E., Pardo G., Tinahones F., Ricart W., Fernández-Real J.M. (2013). Irisin is expressed and produced by human muscle and adipose tissue in association with obesity and insulin resistance. J. Clin. Endocrinol. Metab..

[17] Jedrychowski M.P., Wrann C.D., Paulo J.A., Gerber K.K., Szpyt J., Robinson M.M., Nair K.S., Gygi S.P., Spiegelman B.M. (2015). Detection and quantitation of circulating human irisin by tandem mass spectrometry. Cell Metab..

[18] Raschke S., Elsen M., Gassenhuber H., Sommerfeld M., Schwahn U., Brockmann B., Jung R., Wisløff U., Tjønna A.E., Raastad T. (2013). Evidence against a beneficial effect of irisin in humans. PLoS ONE.

[19] Vanin E.F. (1985). Processed pseudogenes: Characteristics and evolution. Annu. Rev. Genet..

[20] Albrecht E., Norheim F., Thiede B., Holen T., Ohashi T., Schering L., Lee S., Brenmoehl J., Thomas S., Drevon C.A. (2015). Irisin–a myth rather than an exercise-inducible myokine. Sci. Rep..

[21] Lee P., Linderman J.D., Smith S., Brychta R.J., Wang J., Idelson C., Perron R.M., Werner C.D., Phan G.Q., Kammula U.S. (2014). Irisin and FGF21 are cold-induced endocrine activators of brown fat function in humans. Cell Metab..

[22] Ruan Q., Zhang L., Ruan J., Zhang X., Chen J., Ma C., Yu Z. (2018). Detection and quantitation of irisin in human cerebrospinal fluid by tandem mass spectrometry. Peptides.

[23] Ruan Q., Huang Y., Yang L., Ruan J., Gu W., Zhang X., Zhang Y., Zhang W., Yu Z. (2019). The effects of both age and sex on irisin levels in paired plasma and cerebrospinal fluid in healthy humans. Peptides.

[24] Colaianni G., Errede M., Sanesi L., Notarnicola A., Celi M., Zerlotin R., Storlino G., Pignataro P., Oranger A., Pesce V. (2021). Irisin correlates positively with BMD in a cohort of older adult patients and downregulates the senescent marker p21 in osteoblasts. J. Bone Miner. Res..

[25] Colaianni G., Storlino G., Sanesi L., Colucci S., Grano M. (2020). Myokines and osteokines in the pathogenesis of muscle and bone diseases. Curr. Osteoporos. Rep..

[26] Young M.F., Valaris S., Wrann C.D. (2019). A role for FNDC5/Irisin in the beneficial effects of exercise on the brain and in neurodegenerative diseases. Prog. Cardiovasc. Dis..

[27] Schumacher M.A., Chinnam N., Ohashi T., Shah R.S., Erickson H.P. (2013). The structure of irisin reveals a novel intersubunit β-sheet fibronectin type III (FNIII) dimer: Implications for receptor activation. J. Biol. Chem..

[28] Korta P., Pocheć E. (2019). Glycosylation of thyroid-stimulating hormone receptor. Endokrynol. Pol..

[29] Zhang Y., Li R., Meng Y., Li S., Donelan W., Zhao Y., Qi L., Zhang M., Wang X., Cui T. (2014). Irisin stimulates browning of white adipocytes through mitogen-activated protein kinase p38 MAP kinase and ERK MAP kinase signaling. Diabetes.

[30] Rabiee F., Lachinani L., Ghaedi S., Nasr-Esfahani M.H., Megraw T.L., Ghaedi K. (2020). New insights into the cellular activities of Fndc5/Irisin and its signaling pathways. Cell Biosci..

[31] Liu J., Song N., Huang Y., Chen Y. (2018). Irisin inhibits pancreatic cancer cell growth via the AMPK-mTOR pathway. Sci. Rep..

[32] Shao L., Li H., Chen J., Song H., Zhang Y., Wu F., Wang W., Zhang W., Wang F., Li H. (2017). Irisin suppresses the migration, proliferation, and invasion of lung cancer cells via inhibition of epithelial-to-mesenchymal transition. Biochem. Biophys. Res. Commun..

[33] Lourenco M.V., Frozza R.L., de Freitas G.B., Zhang H., Kincheski G.C., Ribeiro F.C., Gonçalves R.A., Clarke J.R., Beckman D., Staniszewski A. (2019). Exercise-linked FNDC5/irisin rescues synaptic plasticity and memory defects in Alzheimer’s models. Nat. Med..

[34] Ma E.B., Sahar N.E., Jeong M., Huh J.Y. (2019). Irisin exerts inhibitory effect on adipogenesis through regulation of Wnt signaling. Front. Physiol..

[35] Kim H., Wrann C.D., Jedrychowski M., Vidoni S., Kitase Y., Nagano K., Zhou C., Chou J., Parkman V.-J.A., Novick S.J. (2018). Irisin mediates effects on bone and fat via αV integrin receptors. Cell.

[36] Pignataro P., Dicarlo M., Zerlotin R., Zecca C., Dell’Abate M.T., Buccoliero C., Logroscino G., Colucci S., Grano M. (2021). FNDC5/Irisin System in Neuroinflammation and Neurodegenerative Diseases: Update and Novel Perspective. Int. J. Mol. Sci..

[37] Liu Y., Zhu C., Guo J., Chen Y., Meng C. (2020). The neuroprotective effect of irisin in ischemic stroke. Front. Aging Neurosci..

[38] Qiao X., Nie Y., Ma Y., Chen Y., Cheng R., Yin W., Hu Y., Xu W., Xu L. (2016). Irisin promotes osteoblast proliferation and differentiation via activating the MAP kinase signaling pathways. Sci. Rep..

[39] Rosen E.D., Spiegelman B.M. (2006). Adipocytes as regulators of energy balance and glucose homeostasis. Nature.

[40] Wang W., Seale P. (2016). Control of brown and beige fat development. Nat. Rev. Mol. Cell Biol..

[41] Luo Y., Qiao X., Ma Y., Deng H., Xu C.C., Xu L. (2020). Disordered metabolism in mice lacking irisin. Sci. Rep..

[42] Park K.H., Zaichenko L., Brinkoetter M., Thakkar B., Sahin-Efe A., Joung K.E., Tsoukas M.A., Geladari E.V., Huh J.Y., Dincer F. (2013). Circulating irisin in relation to insulin resistance and the metabolic syndrome. J. Clin. Endocrinol. Metab..

[43] Liu S., Du F., Li X., Wang M., Duan R., Zhang J., Wu Y., Zhang Q. (2017). Effects and underlying mechanisms of irisin on the proliferation and apoptosis of pancreatic β cells. PLoS ONE.

[44] Chen N., Li Q., Liu J., Jia S. (2016). Irisin, an exercise-induced myokine as a metabolic regulator: An updated narrative review. Diabetes/Metab. Res. Rev..

[45] Choi Y.-K., Kim M.-K., Bae K.H., Seo H.-A., Jeong J.-Y., Lee W.-K., Kim J.-G., Lee I.-K., Park K.-G. (2013). Serum irisin levels in new-onset type 2 diabetes. Diabetes Res. Clin. Pract..

[46] Liu J.-J., Wong M.D., Toy W.C., Tan C.S., Liu S., Ng X.W., Tavintharan S., Sum C.F., Lim S.C. (2013). Lower circulating irisin is associated with type 2 diabetes mellitus. J. Diabetes Its Complicat..

[47] Zhu D., Wang H., Zhang J., Zhang X., Xin C., Zhang F., Lee Y., Zhang L., Lian K., Yan W. (2015). Irisin improves endothelial function in type 2 diabetes through reducing oxidative/nitrative stresses. J. Mol. Cell. Cardiol..

[48] So J.H., Huang C., Ge M., Cai G., Zhang L., Lu Y., Mu Y. (2017). Intense exercise promotes adult hippocampal neurogenesis but not spatial discrimination. Front. Cell. Neurosci..

[49] Novelle M.G., Contreras C., Romero-Picó A., López M., Diéguez C. (2013). Irisin, two years later. Int. J. Endocrinol..

[50] Wrann C.D., White J.P., Salogiannnis J., Laznik-Bogoslavski D., Wu J., Ma D., Lin J.D., Greenberg M.E., Spiegelman B.M. (2013). Exercise induces hippocampal BDNF through a PGC-1α/FNDC5 pathway. Cell Metab..

[51] Moon H.-S., Dincer F., Mantzoros C.S. (2013). Pharmacological concentrations of irisin increase cell proliferation without influencing markers of neurite outgrowth and synaptogenesis in mouse H19-7 hippocampal cell lines. Metabolism.

[52] Pesce M., Ballerini P., Paolucci T., Puca I., Farzaei M.H., Patruno A. (2020). Irisin and autophagy: First update. Int. J. Mol. Sci..

[53] Xin T., Lu C. (2020). Irisin activates Opa1-induced mitophagy to protect cardiomyocytes against apoptosis following myocardial infarction. Aging.

[54] Asadi Y., Gorjipour F., Behrouzifar S., Vakili A. (2018). Irisin peptide protects brain against ischemic injury through reducing apoptosis and enhancing BDNF in a rodent model of stroke. Neurochem. Res..

[55] Guo P., Jin Z., Wu H., Li X., Ke J., Zhang Z., Zhao Q. (2019). Effects of irisin on the dysfunction of blood–brain barrier in rats after focal cerebral ischemia/reperfusion. Brain Behav..

[56] Peng J., Deng X., Huang W., Yu J.-H., Wang J.-X., Wang J.-P., Yang S.-B., Liu X., Wang L., Zhang Y. (2017). Irisin protects against neuronal injury induced by oxygen-glucose deprivation in part depends on the inhibition of ROS-NLRP3 inflammatory signaling pathway. Mol. Immunol..

[57] Yu Q., Li G., Ding Q., Tao L., Li J., Sun L., Sun X., Yang Y. (2020). Irisin protects brain against ischemia/reperfusion injury through suppressing TLR4/MyD88 pathway. Cerebrovasc. Dis..

[58] Jin Z., Guo P., Li X., Ke J., Wang Y., Wu H. (2019). Neuroprotective effects of irisin against cerebral ischemia/reperfusion injury via Notch signaling pathway. Biomed. Pharmacother..

[59] Li D.-J., Li Y.-H., Yuan H.-B., Qu L.-F., Wang P. (2017). The novel exercise-induced hormone irisin protects against neuronal injury via activation of the Akt and ERK1/2 signaling pathways and contributes to the neuroprotection of physical exercise in cerebral ischemia. Metabolism.

[60] Kim O.Y., Song J. (2018). The role of irisin in Alzheimer’s disease. J. Clin. Med..

[61] Waseem R., Anwar S., Khan S., Shamsi A., Hassan M., Anjum F., Shafie A., Islam A., Yadav D.K. (2021). MAP/Microtubule Affinity Regulating Kinase 4 Inhibitory Potential of Irisin: A New Therapeutic Strategy to Combat Cancer and Alzheimer’s Disease. Int. J. Mol. Sci..

[62] Noda Y., Kuzuya A., Tanigawa K., Araki M., Kawai R., Ma B., Sasakura Y., Maesako M., Tashiro Y., Miyamoto M. (2018). Fibronectin type III domain-containing protein 5 interacts with APP and decreases amyloid β production in Alzheimer’s disease. Mol. Brain.

[63] Li G., Thabane L., Papaioannou A., Ioannidis G., Levine M.A., Adachi J.D. (2017). An overview of osteoporosis and frailty in the elderly. BMC Musculoskelet. Disord..

[64] Colaianni G., Cuscito C., Mongelli T., Oranger A., Mori G., Brunetti G., Colucci S., Cinti S., Grano M. (2014). Irisin enhances osteoblast differentiation in vitro. Int. J. Endocrinol..

[65] Estell E.G., Le P.T., Vegting Y., Kim H., Wrann C., Bouxsein M.L., Nagano K., Baron R., Spiegelman B.M., Rosen C.J. (2020). Irisin directly stimulates osteoclastogenesis and bone resorption in vitro and in vivo. eLife.

[66] Serbest S., Tiftikçi U., Tosun H.B., Kısa Ü. (2017). The irisin hormone profile and expression in human bone tissue in the bone healing process in patients. Med. Sci. Monit. Int. Med. J. Exp. Clin. Res..

[67] Singhal V., Lawson E.A., Ackerman K.E., Fazeli P.K., Clarke H., Lee H., Eddy K., Marengi D.A., Derrico N.P., Bouxsein M.L. (2014). Irisin levels are lower in young amenorrheic athletes compared with eumenorrheic athletes and non-athletes and are associated with bone density and strength estimates. PLoS ONE.

[68] Zhu X., Li X., Wang X., Chen T., Tao F., Liu C., Tu Q., Shen G., Chen J.J. (2021). Irisin deficiency disturbs bone metabolism. J. Cell. Physiol..

[69] Maalouf G.-E., El Khoury D. (2019). Exercise-induced irisin, the fat browning myokine, as a potential anticancer agent. J. Obes..

[70] Gannon N.P., Vaughan R.A., Garcia-Smith R., Bisoffi M., Trujillo K.A. (2015). Effects of the exercise-inducible myokine irisin on malignant and non-malignant breast epithelial cell behavior in vitro. Int. J. Cancer.

[71] Hanahan D., Weinberg R.A. (2011). Hallmarks of cancer: The next generation. Cell.

[72] Provatopoulou X., Georgiou G.P., Kalogera E., Kalles V., Matiatou M.A., Papapanagiotou I., Sagkriotis A., Zografos G.C., Gounaris A. (2015). Serum irisin levels are lower in patients with breast cancer: Association with disease diagnosis and tumor characteristics. BMC Cancer.

[73] Pinkowska A., Podhorska-Okołów M., Dzięgiel P., Nowińska K. (2021). The Role of Irisin in Cancer Disease. Cells.

[74] Kong G., Jiang Y., Sun X., Cao Z., Zhang G., Zhao Z., Zhao Y., Yu Q., Cheng G. (2017). Irisin reverses the IL-6 induced epithelial-mesenchymal transition in osteosarcoma cell migration and invasion through the STAT3/Snail signaling pathway. Oncol. Rep..

[75] Altay D.U., Keha E.E., Karagüzel E., Menteşe A., Yaman S.O., Alver A. (2018). The diagnostic value of FNDC5/Irisin in renal Cell Cancer. Int. Braz. J. Urol..

[76] Waseem R., Shamsi A., Shahbaz M., Khan T., Kazim S.N., Ahmad F., Hassan M.I., Islam A. (2022). Effect of pH on the structure and stability of irisin, a multifunctional protein: Multispectroscopic and molecular dynamics simulation approach. J. Mol. Struct..

[77] Lanni C., Masi M., Racchi M., Govoni S. (2021). Cancer and Alzheimer’s disease inverse relationship: An age-associated diverging derailment of shared pathways. Mol. Psychiatry.

